# Supplementary material for: Metabolomic signatures in adults with metabolic syndrome indicate preclinical disruptions in pathways associated with high-density lipoprotein cholesterol, sugar alcohols
Source: Cardiovasc Diabetol Endocrinol Rep. 2025 Jun 10;11:11. doi: 10.1186/s40842-025-00223-x (PMC12150447; doi:10.1186/s40842-025-00223-x)
Supplement: Supplementary file 2 — Supplementary Material 2. Supplementary Table 2. Metabolites Significantly Associated with Baseline Lipid Clinical Characteristics (LDL, HDL, TG), q < 0.05 [file 40842_2025_223_MOESM2_ESM.docx]

Supplementary Table 2. Metabolites Significantly Associated with Baseline Lipid Clinical Characteristics (LDL, HDL, TG), *q* < 0.05

| **Metabolite** | **LDL**  ***r*** | **p.**  **LDL** | **FDR.LDL** | **HDL**  ***r*** | **p.**  **HDL** | **FDR.**  **HDL** | **TG**  ***r*** | **p.**  **TG** | **FDR.**  **TG** |
| --- | --- | --- | --- | --- | --- | --- | --- | --- | --- |
| 1-methyl galactose | -0.08 | 0.39 | 0.89 | 0.04 | 0.69 | 0.84 | -0.09 | 0.31 | 0.73 |
| 1-monopalmitin | 0.04 | 0.65 | 0.89 | -0.08 | 0.39 | 0.66 | 0.25 | 0.01 | 0.51 |
| 1-monostearin | -0.06 | 0.52 | 0.89 | -0.08 | 0.40 | 0.66 | 0.10 | 0.25 | 0.69 |
| 2,3-dihydroxy-butanoic acid | 0.08 | 0.37 | 0.89 | -0.25 | 0.01 | 0.07 | 0.12 | 0.19 | 0.63 |
| 2,3-dihydroxy-pyridine | 0.06 | 0.53 | 0.89 | -0.12 | 0.18 | 0.40 | 0.01 | 0.94 | 0.97 |
| 2-aminobutyric acid | 0.11 | 0.21 | 0.89 | -0.23 | 0.01 | 0.08 | 0.00 | 0.96 | 0.97 |
| 2-deoxytetronic acid | 0.13 | 0.14 | 0.89 | -0.01 | 0.93 | 0.98 | -0.06 | 0.49 | 0.90 |
| 2-hydroxybutanoic acid | -0.07 | 0.42 | 0.89 | -0.05 | 0.60 | 0.78 | 0.00 | 0.98 | 0.98 |
| 2-hydroxyglutaric acid | 0.05 | 0.55 | 0.89 | 0.13 | 0.14 | 0.34 | -0.03 | 0.74 | 0.92 |
| 2-hydroxyhippuric acid | -0.10 | 0.29 | 0.89 | 0.05 | 0.59 | 0.78 | -0.21 | 0.02 | 0.54 |
| 2-hydroxyisovaleric acid | 0.12 | 0.19 | 0.89 | -0.25 | 0.00 | 0.07 | 0.17 | 0.06 | 0.63 |
| 2-hydroxyvaleric acid | -0.09 | 0.31 | 0.89 | -0.19 | 0.04 | 0.14 | 0.22 | 0.01 | 0.51 |
| 2-ketoisocaproic acid | 0.00 | 0.97 | 1.00 | -0.24 | 0.01 | 0.07 | 0.16 | 0.08 | 0.63 |
| 2-picolinic acid | -0.10 | 0.28 | 0.89 | -0.03 | 0.75 | 0.90 | 0.09 | 0.29 | 0.72 |
| 3-Aminopiperidine-2,6-dione | 0.18 | 0.04 | 0.80 | -0.10 | 0.28 | 0.54 | 0.12 | 0.19 | 0.63 |
| 3-hydroxybutyric acid | 0.06 | 0.48 | 0.89 | -0.09 | 0.33 | 0.59 | -0.01 | 0.93 | 0.97 |
| 9-myristoleate | 0.08 | 0.36 | 0.89 | 0.05 | 0.61 | 0.78 | 0.04 | 0.62 | 0.92 |
| Aconitic acid | 0.09 | 0.33 | 0.89 | -0.10 | 0.27 | 0.51 | 0.05 | 0.60 | 0.92 |
| Adipic acid | 0.07 | 0.41 | 0.89 | -0.17 | 0.05 | 0.18 | 0.05 | 0.60 | 0.92 |
| Alanine | -0.06 | 0.49 | 0.89 | -0.05 | 0.61 | 0.78 | 0.12 | 0.18 | 0.63 |
| Allantoic acid | -0.06 | 0.53 | 0.89 | -0.04 | 0.66 | 0.81 | -0.04 | 0.63 | 0.92 |
| Alloxanoic acid | 0.06 | 0.50 | 0.89 | -0.07 | 0.41 | 0.66 | 0.05 | 0.57 | 0.92 |
| Anthranilic acid | -0.20 | 0.02 | 0.73 | -0.07 | 0.43 | 0.66 | -0.11 | 0.21 | 0.63 |
| Arabitol | -0.04 | 0.69 | 0.89 | 0.02 | 0.85 | 0.96 | -0.04 | 0.70 | 0.92 |
| Arachidic acid | 0.10 | 0.26 | 0.89 | 0.00 | 0.96 | 0.99 | 0.13 | 0.16 | 0.63 |
| Arachidonic acid | 0.01 | 0.88 | 1.00 | 0.06 | 0.53 | 0.74 | -0.12 | 0.18 | 0.63 |
| Asparagine | 0.09 | 0.31 | 0.89 | -0.18 | 0.05 | 0.17 | 0.01 | 0.89 | 0.97 |
| Aspartate | 0.13 | 0.16 | 0.89 | -0.25 | 0.00 | 0.07 | 0.16 | 0.07 | 0.63 |
| Azelaic acid | -0.01 | 0.92 | 1.00 | -0.11 | 0.22 | 0.46 | 0.06 | 0.53 | 0.92 |
| Benzoic acid | 0.03 | 0.71 | 0.90 | -0.11 | 0.22 | 0.46 | -0.01 | 0.95 | 0.97 |
| Beta alanine | 0.11 | 0.21 | 0.89 | -0.09 | 0.29 | 0.54 | 0.02 | 0.81 | 0.95 |
| Biuret | -0.11 | 0.24 | 0.89 | -0.14 | 0.12 | 0.32 | 0.08 | 0.37 | 0.79 |
| Butane-2,3-diol | -0.06 | 0.50 | 0.89 | -0.01 | 0.90 | 0.97 | 0.08 | 0.39 | 0.79 |
| Capric acid | 0.08 | 0.38 | 0.89 | -0.10 | 0.26 | 0.51 | 0.10 | 0.28 | 0.71 |
| Caproic acid | 0.23 | 0.01 | 0.49 | -0.25 | 0.01 | 0.07 | 0.08 | 0.39 | 0.79 |
| Cholesterol | 0.19 | 0.03 | 0.73 | -0.21 | 0.02 | 0.10 | 0.14 | 0.11 | 0.63 |
| Cholic acid | 0.00 | 0.99 | 1.00 | -0.17 | 0.06 | 0.20 | -0.04 | 0.66 | 0.92 |
| Citric acid | 0.03 | 0.74 | 0.93 | -0.07 | 0.41 | 0.66 | -0.01 | 0.95 | 0.97 |
| Citrulline | 0.01 | 0.90 | 1.00 | -0.15 | 0.09 | 0.27 | -0.02 | 0.83 | 0.95 |
| Conduritol-beta-epoxide | 0.08 | 0.37 | 0.89 | -0.15 | 0.09 | 0.27 | -0.03 | 0.73 | 0.92 |
| Creatinine | -0.03 | 0.71 | 0.90 | -0.16 | 0.08 | 0.26 | 0.02 | 0.87 | 0.97 |
| Cystine | -0.08 | 0.36 | 0.89 | 0.00 | 0.98 | 0.99 | 0.05 | 0.58 | 0.92 |
| Deoxypentitol | 0.02 | 0.85 | 1.00 | -0.14 | 0.11 | 0.29 | 0.03 | 0.72 | 0.92 |
| Dopamine | 0.07 | 0.46 | 0.89 | 0.00 | 0.96 | 0.99 | 0.12 | 0.20 | 0.63 |
| Erythritol | -0.09 | 0.32 | 0.89 | -0.09 | 0.31 | 0.57 | -0.01 | 0.90 | 0.97 |
| Ethanolamine | -0.02 | 0.86 | 1.00 | -0.11 | 0.21 | 0.45 | -0.04 | 0.67 | 0.92 |
| Fructose | 0.02 | 0.83 | 0.99 | 0.06 | 0.47 | 0.68 | -0.22 | 0.01 | 0.51 |
| Fucose | -0.12 | 0.20 | 0.89 | -0.02 | 0.83 | 0.95 | -0.06 | 0.52 | 0.92 |
| Fumaric acid | -0.02 | 0.79 | 0.97 | -0.15 | 0.08 | 0.27 | 0.05 | 0.58 | 0.92 |
| Gluconic acid | 0.04 | 0.66 | 0.89 | -0.20 | 0.02 | 0.10 | 0.04 | 0.64 | 0.92 |
| Gluconic acid- lactone | 0.00 | 0.99 | 1.00 | -0.24 | 0.01 | 0.07 | -0.14 | 0.13 | 0.63 |
| Glucose | 0.01 | 0.89 | 1.00 | -0.19 | 0.03 | 0.13 | 0.03 | 0.72 | 0.92 |
| Glucose-1-phosphate | -0.05 | 0.56 | 0.89 | -0.19 | 0.03 | 0.12 | 0.01 | 0.95 | 0.97 |
| Glutamate | 0.07 | 0.46 | 0.89 | -0.24 | 0.01 | 0.07 | 0.12 | 0.20 | 0.63 |
| Glutamine | 0.04 | 0.67 | 0.89 | -0.15 | 0.09 | 0.27 | -0.05 | 0.59 | 0.92 |
| Glutaric acid | 0.14 | 0.13 | 0.89 | -0.03 | 0.73 | 0.87 | 0.03 | 0.78 | 0.94 |
| Glyceric acid | 0.14 | 0.12 | 0.89 | 0.07 | 0.44 | 0.66 | 0.04 | 0.67 | 0.92 |
| Glycerol | -0.06 | 0.50 | 0.89 | 0.21 | 0.02 | 0.09 | -0.03 | 0.72 | 0.92 |
| Glycerol-3-galactoside | 0.04 | 0.65 | 0.89 | -0.07 | 0.41 | 0.66 | -0.03 | 0.74 | 0.92 |
| Glycerol-alpha-phosphate | 0.12 | 0.17 | 0.89 | -0.07 | 0.43 | 0.66 | 0.13 | 0.15 | 0.63 |
| Glycine | -0.01 | 0.88 | 1.00 | 0.06 | 0.48 | 0.68 | -0.19 | 0.04 | 0.55 |
| Glycolic acid | -0.07 | 0.42 | 0.89 | 0.06 | 0.47 | 0.68 | 0.04 | 0.67 | 0.92 |
| Heptadecanoic acid | 0.00 | 0.97 | 1.00 | -0.24 | 0.01 | 0.07 | 0.15 | 0.10 | 0.63 |
| Heptanoic acid | 0.05 | 0.56 | 0.89 | -0.15 | 0.10 | 0.28 | 0.03 | 0.75 | 0.92 |
| Hydroxycarbamate | 0.08 | 0.38 | 0.89 | -0.08 | 0.38 | 0.66 | -0.05 | 0.55 | 0.92 |
| Hypoxanthine | -0.02 | 0.80 | 0.98 | -0.02 | 0.82 | 0.95 | -0.13 | 0.16 | 0.63 |
| Indole-3-acetate | -0.10 | 0.27 | 0.89 | 0.09 | 0.33 | 0.59 | 0.07 | 0.43 | 0.81 |
| Indole-3-lactate | 0.08 | 0.40 | 0.89 | -0.24 | 0.01 | 0.07 | 0.16 | 0.07 | 0.63 |
| Indole-3-propionic acid | -0.09 | 0.34 | 0.89 | 0.10 | 0.25 | 0.49 | -0.17 | 0.06 | 0.63 |
| Isocitric acid | 0.06 | 0.50 | 0.89 | -0.04 | 0.63 | 0.80 | 0.09 | 0.34 | 0.76 |
| Isoheptadecanoic acid | 0.01 | 0.91 | 1.00 | -0.21 | 0.02 | 0.09 | 0.10 | 0.28 | 0.71 |
| Isoleucine | -0.04 | 0.68 | 0.89 | -0.33 | 0.00 | 0.01 | 0.13 | 0.15 | 0.63 |
| Isooctanol | -0.09 | 0.34 | 0.89 | -0.07 | 0.46 | 0.68 | 0.12 | 0.18 | 0.63 |
| Isothreonic acid | 0.17 | 0.06 | 0.89 | 0.00 | 0.96 | 0.99 | 0.08 | 0.37 | 0.79 |
| Lactic acid | -0.05 | 0.55 | 0.89 | -0.06 | 0.51 | 0.71 | 0.08 | 0.38 | 0.79 |
| Lactose | -0.04 | 0.62 | 0.89 | 0.01 | 0.87 | 0.97 | 0.01 | 0.93 | 0.97 |
| Lauric acid | -0.05 | 0.55 | 0.89 | 0.01 | 0.94 | 0.98 | 0.03 | 0.73 | 0.92 |
| Leucine | 0.00 | 0.98 | 1.00 | -0.33 | 0.00 | 0.01 | 0.12 | 0.16 | 0.63 |
| Levoglucosan | -0.05 | 0.60 | 0.89 | -0.19 | 0.03 | 0.13 | 0.04 | 0.63 | 0.92 |
| Linoleic acid | 0.08 | 0.35 | 0.89 | 0.01 | 0.87 | 0.97 | 0.09 | 0.31 | 0.73 |
| Lysine | -0.04 | 0.67 | 0.89 | -0.14 | 0.12 | 0.32 | -0.04 | 0.66 | 0.92 |
| Lyxitol | 0.12 | 0.20 | 0.89 | 0.01 | 0.92 | 0.98 | 0.11 | 0.22 | 0.64 |
| Maleic acid | -0.04 | 0.62 | 0.89 | -0.17 | 0.05 | 0.18 | 0.13 | 0.16 | 0.63 |
| Malic acid | -0.12 | 0.17 | 0.89 | -0.04 | 0.69 | 0.83 | 0.00 | 0.98 | 0.98 |
| Malonic acid | 0.02 | 0.82 | 0.99 | -0.05 | 0.60 | 0.78 | -0.08 | 0.36 | 0.79 |
| Maltose | 0.08 | 0.37 | 0.89 | -0.01 | 0.89 | 0.97 | 0.02 | 0.82 | 0.95 |
| Maltotriose | 0.05 | 0.55 | 0.89 | 0.06 | 0.51 | 0.71 | 0.03 | 0.71 | 0.92 |
| Mannose | 0.02 | 0.79 | 0.97 | -0.29 | 0.00 | 0.03 | -0.12 | 0.19 | 0.63 |
| Methanol phosphate | 0.10 | 0.28 | 0.89 | -0.15 | 0.10 | 0.28 | 0.13 | 0.14 | 0.63 |
| Methionine | 0.10 | 0.25 | 0.89 | -0.25 | 0.01 | 0.07 | 0.12 | 0.19 | 0.63 |
| Methionine sulfoxide | -0.06 | 0.48 | 0.89 | -0.11 | 0.23 | 0.46 | -0.14 | 0.11 | 0.63 |
| Myoinositol | 0.04 | 0.66 | 0.89 | -0.05 | 0.56 | 0.77 | -0.07 | 0.41 | 0.80 |
| Myristic acid | 0.04 | 0.66 | 0.89 | -0.01 | 0.89 | 0.97 | 0.09 | 0.30 | 0.73 |
| N-acetyl-ornithine | 0.01 | 0.87 | 1.00 | -0.21 | 0.02 | 0.10 | 0.08 | 0.39 | 0.79 |
| N-formyl-glycine | -0.05 | 0.60 | 0.89 | 0.00 | 0.99 | 0.99 | -0.06 | 0.50 | 0.90 |
| Nicotinic acid | 0.09 | 0.30 | 0.89 | -0.16 | 0.08 | 0.26 | -0.10 | 0.27 | 0.70 |
| Octanoic acid | 0.07 | 0.42 | 0.89 | -0.23 | 0.01 | 0.08 | 0.09 | 0.33 | 0.75 |
| Oleic acid | 0.07 | 0.43 | 0.89 | 0.05 | 0.57 | 0.77 | 0.12 | 0.19 | 0.63 |
| Ornithine | -0.08 | 0.40 | 0.89 | -0.22 | 0.01 | 0.08 | -0.02 | 0.83 | 0.95 |
| Oxalic acid | 0.19 | 0.04 | 0.73 | -0.01 | 0.90 | 0.97 | -0.13 | 0.14 | 0.63 |
| Oxoproline | 0.05 | 0.56 | 0.89 | -0.21 | 0.02 | 0.10 | 0.06 | 0.53 | 0.92 |
| Palmitic acid | 0.04 | 0.68 | 0.89 | -0.10 | 0.25 | 0.49 | 0.19 | 0.04 | 0.55 |
| Palmitoleic acid | 0.11 | 0.24 | 0.89 | 0.22 | 0.01 | 0.09 | 0.01 | 0.91 | 0.97 |
| Pelargonic acid | -0.01 | 0.87 | 1.00 | -0.05 | 0.55 | 0.75 | 0.08 | 0.36 | 0.79 |
| Phenol | 0.05 | 0.62 | 0.89 | -0.13 | 0.16 | 0.37 | 0.12 | 0.18 | 0.63 |
| Phenylalanine | 0.05 | 0.61 | 0.89 | -0.20 | 0.02 | 0.10 | 0.05 | 0.59 | 0.92 |
| Phenylethylamine | 0.09 | 0.30 | 0.89 | -0.14 | 0.13 | 0.32 | -0.13 | 0.16 | 0.63 |
| Phosphate | 0.10 | 0.27 | 0.89 | -0.08 | 0.39 | 0.66 | -0.02 | 0.81 | 0.95 |
| Phosphor-ethanolamine | 0.01 | 0.93 | 1.00 | -0.01 | 0.89 | 0.97 | 0.01 | 0.89 | 0.97 |
| Phthalic acid | -0.06 | 0.48 | 0.89 | -0.15 | 0.10 | 0.28 | -0.03 | 0.75 | 0.92 |
| Proline | -0.05 | 0.59 | 0.89 | -0.23 | 0.01 | 0.08 | 0.12 | 0.18 | 0.63 |
| Propane-1,3-diol | -0.06 | 0.52 | 0.89 | -0.21 | 0.02 | 0.09 | 0.05 | 0.55 | 0.92 |
| Propylamine | -0.07 | 0.47 | 0.89 | -0.08 | 0.37 | 0.66 | 0.02 | 0.87 | 0.97 |
| Pseudouridine | 0.06 | 0.51 | 0.89 | -0.05 | 0.61 | 0.78 | 0.07 | 0.41 | 0.80 |
| Putrescine | 0.06 | 0.47 | 0.89 | -0.04 | 0.67 | 0.82 | -0.01 | 0.90 | 0.97 |
| Pyrophosphate | 0.07 | 0.46 | 0.89 | 0.02 | 0.83 | 0.95 | -0.05 | 0.61 | 0.92 |
| Quinic acid | -0.04 | 0.69 | 0.89 | -0.08 | 0.38 | 0.66 | -0.07 | 0.47 | 0.87 |
| Ribitol | 0.00 | 1.00 | 1.00 | -0.13 | 0.14 | 0.34 | 0.10 | 0.27 | 0.70 |
| Ribonic acid | -0.12 | 0.17 | 0.89 | -0.07 | 0.42 | 0.66 | -0.01 | 0.93 | 0.97 |
| Ribose | -0.17 | 0.06 | 0.89 | -0.13 | 0.16 | 0.37 | 0.12 | 0.20 | 0.63 |
| Saccharic acid | 0.13 | 0.14 | 0.89 | -0.06 | 0.49 | 0.70 | 0.05 | 0.56 | 0.92 |
| Salicylic acid | -0.11 | 0.24 | 0.89 | 0.00 | 0.96 | 0.99 | -0.18 | 0.05 | 0.61 |
| Serine | 0.08 | 0.38 | 0.89 | -0.10 | 0.25 | 0.49 | -0.11 | 0.20 | 0.63 |
| Shikimic acid | 0.11 | 0.20 | 0.89 | -0.08 | 0.40 | 0.66 | 0.05 | 0.60 | 0.92 |
| Stearic acid | 0.01 | 0.94 | 1.00 | -0.24 | 0.01 | 0.07 | 0.17 | 0.05 | 0.63 |
| Stigmasterol | 0.16 | 0.07 | 0.89 | -0.21 | 0.02 | 0.09 | 0.03 | 0.72 | 0.92 |
| Suberic acid | 0.00 | 0.98 | 1.00 | -0.14 | 0.12 | 0.32 | -0.07 | 0.42 | 0.80 |
| Succinic acid | -0.04 | 0.68 | 0.89 | -0.12 | 0.17 | 0.39 | 0.19 | 0.03 | 0.55 |
| Sucrose | 0.03 | 0.75 | 0.93 | -0.11 | 0.20 | 0.44 | 0.07 | 0.46 | 0.87 |
| Terephthalic acid | 0.00 | 0.96 | 1.00 | -0.14 | 0.12 | 0.32 | 0.03 | 0.73 | 0.92 |
| Threitol | 0.04 | 0.69 | 0.89 | 0.16 | 0.08 | 0.26 | -0.10 | 0.28 | 0.71 |
| Threonic acid | -0.16 | 0.08 | 0.89 | 0.07 | 0.42 | 0.66 | -0.23 | 0.01 | 0.51 |
| Threonine | 0.01 | 0.93 | 1.00 | -0.21 | 0.02 | 0.10 | -0.11 | 0.22 | 0.64 |
| Tocopherol-alpha | 0.33 | 0.00 | 0.03 | -0.04 | 0.65 | 0.81 | 0.20 | 0.02 | 0.55 |
| Tocopherol-gamma | 0.19 | 0.03 | 0.73 | -0.13 | 0.16 | 0.37 | 0.04 | 0.66 | 0.92 |
| Trans-4-Hydroxyproline | -0.11 | 0.22 | 0.89 | -0.13 | 0.16 | 0.37 | 0.02 | 0.83 | 0.95 |
| Trehalose | 0.12 | 0.18 | 0.89 | -0.03 | 0.77 | 0.91 | -0.01 | 0.89 | 0.97 |
| Tryptophan | -0.10 | 0.28 | 0.89 | -0.20 | 0.02 | 0.10 | 0.14 | 0.11 | 0.63 |
| Tyrosine | 0.01 | 0.90 | 1.00 | -0.23 | 0.01 | 0.08 | 0.02 | 0.83 | 0.95 |
| Urea | 0.08 | 0.36 | 0.89 | -0.20 | 0.02 | 0.10 | 0.12 | 0.17 | 0.63 |
| Uric Acid | -0.01 | 0.95 | 1.00 | -0.19 | 0.03 | 0.13 | 0.19 | 0.03 | 0.55 |
| Uridine | 0.23 | 0.01 | 0.49 | 0.00 | 0.98 | 0.99 | 0.11 | 0.24 | 0.67 |
| Valine | 0.00 | 1.00 | 1.00 | -0.30 | 0.00 | 0.03 | 0.11 | 0.21 | 0.63 |
| Xylitol | 0.08 | 0.39 | 0.89 | 0.07 | 0.44 | 0.66 | 0.11 | 0.23 | 0.65 |
| Xylose | -0.07 | 0.44 | 0.89 | 0.03 | 0.78 | 0.91 | -0.05 | 0.61 | 0.92 |

FDR=False Discovery Rate; LDL=Low Density Lipoprotein Cholesterol; HDL=High Density Lipoprotein Cholesterol; TG=Triglycerides
